# Evaluation of Thermal Stability and Thermal Transitions of Hydroxyl-Terminated Polysiloxane/Montmorillonite Nanocomposites

**DOI:** 10.3390/ma18061226

**Published:** 2025-03-10

**Authors:** Sozon P. Vasilakos, Petroula A. Tarantili

**Affiliations:** Polymer Technology Lab., School of Chemical Engineering, National Technical University of Athens, 15780 Athens, Greece; sozon_vas@hotmail.com

**Keywords:** polydimethylsiloxane, montmorillonite, X-ray diffraction, differential scanning calorimetry, thermogravimetric analysis

## Abstract

Condensation-type polysiloxane composites with montmorillonite (MMT) of different organic modifications were prepared in this study. X-ray diffraction (XRD) characterization revealed that the higher degree of organic modification in Cloisite 20A, compared to that in Cloisite 30B, resulted in a larger interlayer spacing between the clay platelets. This facilitates the insertion of elastomer chains between the layers, enabling easier exfoliation and dispersion in the elastomeric matrix. Differential scanning calorimetry (DSC) showed that the reinforcing agents used reduced the cold crystallization temperature of the condensation-type polysiloxane while leaving the glass transition and melting temperatures nearly unaffected. Additionally, the nanocomposites exhibited slightly lower crystallization and melting enthalpies compared to pure silicone. Thermogravimetric analysis (TGA) showed that incorporating the two organically modified clays (Cloisite 20A and Cloisite 30B) into the condensation-type polysiloxane significantly improved the thermal stability of the resulting nanocomposites. This improvement was reflected in the significant increase in the onset and maximum degradation rate temperatures across all examined reinforcement ratios. It was observed that a higher degree of organic modification in MMT (Cloisite 20A) resulted in a more efficient dispersion in the PDMS matrix and enhanced the thermal stability of the composites. These PDMS nanocomposites could be suitable as protective coatings for devices exposed to elevated temperatures.

## 1. Introduction

Organoclays have been extensively studied for the preparation of polymer-matrix nanocomposites. The organic modification of clay minerals improves clay/polymer interactions and ensures uniform dispersion within the polymer matrix. A significant amount of research has been conducted to correlate clay intercalation/exfoliation with the properties of their composites.

Many studies have concluded that the presence of nanoparticles does not significantly affect the crystallinity of the composites [1]. Clay layers, on one hand, act as effective heterogeneous nucleation agents, and on the other, restrict the mobility of the chains, reducing the crystallization rate [2]. Therefore, the effect of the reinforcing medium in nanocomposites results from the combined action of these conflicting factors.

Regarding the thermal stability of nanocomposites, it has been shown that the actual decomposition rate of a polymer composite depends on the morphology of the sample [1]. The formation of a nanostructure enhances the thermal stability of the sample, as the dispersed clay particles create a labyrinthine path that hinders diffusion, blocking the transport of volatile decomposition products, and slowing the inward flow of oxygen and heat. However, depending on its amount, clay may cause premature extensive scission of the polymer chains, shifting the decomposition temperature of the matrix to lower values [3].

Wang et al. [2], studying the thermal degradation of unreinforced room-temperature vulcanized condensation silicone and its nanocomposites with OMMT at concentrations of 5, 10, and 20 phr, concluded that the onset temperature and maximum decomposition rate increased for of 5 and 10 phr. However, at 20 phr, the temperatures decreased. This initial increase and subsequent decrease were attributed to an increase in the amount of organic modification of the reinforcing agent. The temperature at the maximum decomposition rate was influenced by the amount of silicone that penetrated the silicate layers. The more silicone chains protected by the OMMT layers, the more thermally resistant the sample becomes, increasing the temperature at which the maximum rate occurs.

Similar findings were reported by Yuan et al. [4], who studied the effect of organically modified montmorillonite on phenyl-methyl-polysiloxane. For concentrations above 8 phr, the reduction in the thermal properties was attributed to the increased amount of organically modified montmorillonite nanoparticles and their heterogeneous dispersion.

Burnside and Giannelis [5] observed that the introduction of organically modified montmorillonite (10% SO4682) improves the thermal stability of unreinforced condensation polysiloxane by increasing the onset and maximum decomposition rate temperatures. This increase was attributed to the reduced permeability of the nanoparticles, which hinders the removal of volatile decomposition products [6,7].

Wang et al. [8], in their study on the thermal stability of condensation polysiloxane nanocomposites with organically modified montmorillonite, found an improvement in the onset temperature, maximum decomposition rate, and combustion residue. This was attributed to two factors: (a) inactivation of the active chain centers of the elastomer, which decompose due to physical contact with the montmorillonite layers, and (b) increased physical and chemical interactions between the layers and polymer chains, which protect the polymer from thermal degradation [9].

Lewicki et al. [10] reported a decrease in the thermal stability of condensation polysiloxane nanocomposites reinforced with organically modified montmorillonite, as they showed a lower onset decomposition temperature, though they noted a reduction in the release rate of volatiles from the nanocomposite mass.

In another study, Wang et al. [11] found that increasing the amount of organically modified montmorillonite using N,N-di(2-hydroxyethyl)-N-dodecyl-N-methylammonium chloride as an intercalation agent in a polysiloxane matrix reduced the combustion residue, with the onset and maximum decomposition rate temperatures showing an initial increase and then a decrease compared to the unreinforced elastomer.

Kim et al. [12] observed a slight increase in thermal stability in addition polysiloxane composites (vulcanized at high temperature) reinforced with both modified and unmodified montmorillonite nanoparticles. They concluded that the parameter that improved the nanocomposite’s thermal stability is the dispersion of the reinforcing medium within the polymer matrix. 

Ismail et al. [13] mentioned that the thermal stability in air of silicone rubber (SR) was strongly dependent on the clay morphology and increased in the following order: highly intercalated/exfoliated Cloisite Na^+^ (SR/Na^+^MMT) < highly intercalated Cloisite 20A (SR/C20A) < highly exfoliated Cloisite 30B (SR/C30B). This effect was further supported by a substantial increase in the final residue of the SR/C30B nanocomposites after TGA, compared to neat SR, as well as by the higher activation energy of decomposition.

TGA analysis in air, from room temperature to 250 °C, of hydroxyl-terminated PDMS reinforced with 2% *w*/*w* organoclay (Cloisite 30B) revealed a marginal enhancement in the initial decomposition temperature and char residue compared to unmodified PDMS [14].

Wójcik-Bania [15] studied the effect of varying amounts of organo-montmorillonite (0, 1, 2, 4, and 8 wt. %) on a polysiloxane matrix prepared by cross-linking poly (methylhydrosiloxane) with linear divinylsiloxane through a hydrosilylation reaction. It was observed that increasing the amount of organo-montmorillonite led to a deterioration in the thermal stability of the nanocomposites, an increase in the number of thermal decomposition steps, and changes in the degradation mechanism of the polysiloxane matrix.

In this work, we attempted to correlate the organoclay/PDMS nanocomposite structure with the thermal transitions and thermal stability of the hydroxyl-terminated PDMS matrix. In this respect, the effect of the organic surfactant used for the modification of montmorillonite on the thermal transitions and thermal degradation behavior of PDMS nanocomposites in an inert atmosphere was examined.

## 2. Materials and Methods

### 2.1. Materials

Silanol-terminated PDMS (grade DMS-S35, Gelest Inc., Morrisville, PA, USA) with a molecular weight (MW) of 49,000 g/mol was the silicon-base elastomer used in this work. The vulcanization reaction system consisted of 10 phr tetrapropoxysilane (TPOS, Sigma-Aldrich, St. Louis, MO, USA) as a cross-linker and 0.1 phr dibutyl tin dilaurate (Sigma-Aldrich, St. Louis, MO, USA) as a catalyst. Commercial montmorillonite clays, under the trade names Nanofil^®^ 116 [Nan116], Cloisite^®^ 20A [Cl20A], and Cloisite^®^ 30B [Cl30B], were supplied by Rockwood Clay Additives GmbH (Moosburg, Germany). The main characteristics of the organoclays are presented in Table 1.

### 2.2. Preparation of Nanocomposites

Efficient dispersion of the nanoparticles was achieved by sonicating the appropriate amount of clay for 6 min at room temperature, using an ultrasound probe, the PDMS. The cross-linking agents were then added and dispersed into the mixture, and the samples were cast into molds and cured at room temperature for 12 h.

### 2.3. X-Ray Diffractometer (XRD)

X-ray diffraction (XRD) of clay and nanocomposites was performed in order to detect the evolution of the clay d_001_ reflection. A Siemens 5000 apparatus (35 kV, 25 mA) was used (Siemens, Karlsruhe, Germany), employing CuKα Χ-ray radiation with a wavelength of λ = 0.154 nm. The diffractograms were scanned in the 2θ range from 2–10°, at a rate of 2°/min.

XRD characterization is based on the fact that the regular layered structure of clay minerals allows the determination of the interlayer thickness, d, using Bragg’s law:

nλ = 2dsinθ(1)

where: n is an integer (order of reflection), λ is the wavelength of the X-rays and θ is the diffraction angle.

### 2.4. Scanning Electron Microscopy (SEM)

SEM micrographs were obtained from gold-coated, cryogenically fractured surfaces by using a JEOL 2000 scanning electron microscope (JEOL, Tokyo, Japan). 

### 2.5. Differential Scanning Calorimetry (DSC)

DSC measurements were performed using a DSC 1 model Mettler Toledo differential scanning calorimeter (Mettler Toledo, Schwerzenbach, Switzerland). Samples of approximately 10 mg were accurately weighed using an analytical balance and encapsulated in aluminum pans. All runs were conducted under a nitrogen flow of 20 cm^3^/min to limit thermo-oxidative degradation. The samples were cooled from 30 to −150 °C at a rate of −10 °C/min and remained at this temperature for 5 min to erase the previous thermal history. After this treatment, the samples were heated from −150 to 30 °C at 5 °C/min. All tests were conducted in triplicate. The temperatures of glass transition (*T_g_*), crystallization (*T_c_*) and melting (*T_m_*), as well as the heat of fusion (Δ*H_m_*), were calculated from the thermographs corresponding to the heating cycle.

### 2.6. Thermogravimetric Analysis (TGA)

TGA measurements were performed using a Mettler Toledo (TGA-DTA model) thermal gravimetric analyzer (Mettler Toledo, Schwerzenbach, Switzerland). The tests were run with samples of 8–10 mg from 25 °C to 800 °C, at a heating rate of 10 °C/min, under nitrogen atmosphere. The tests were conducted in triplicate.

## 3. Results

### 3.1. X-Ray Diffraction (XRD)

The nanoparticles of Cloisite 20A exhibit three peaks, corresponding to 2θ angles of 3.93°, 5.16°, and 7.57°, while the nanoparticles of Cloisite 30B show a single peak corresponding to a 2θ angle of 5.07°. 

**Figure 1:**
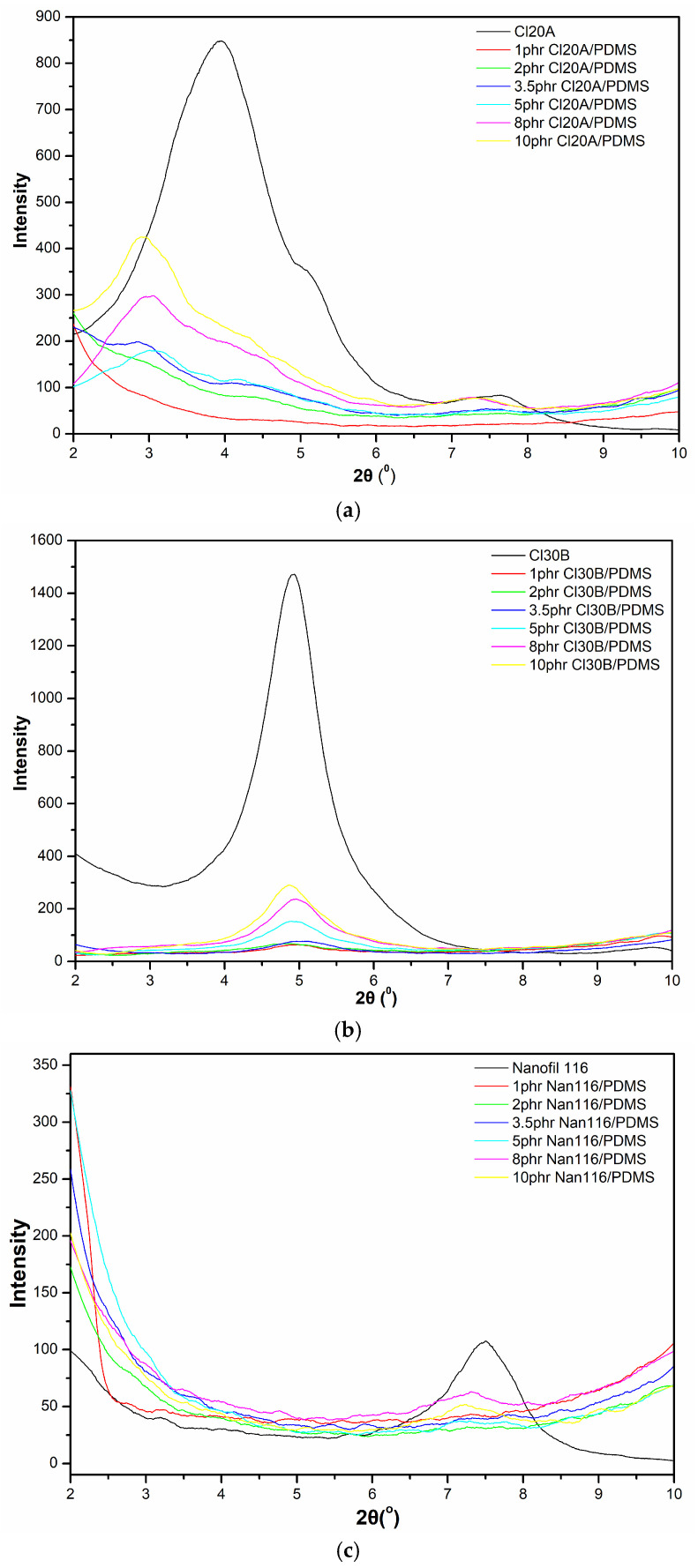
XRD patterns of nanocomposites: (**a**) Cloisite 20A/PDMS, (**b**) Cloisite 30B/PDMS, and (**c**) Nanofil 116/PDMS.

From the X-ray diffraction pattern (Figure 1a) and Table 2, it is evident that for Cloisite 20A concentrations of 1 and 2 phr, no diffraction peak is observed. In contrast, at higher ratios, a peak shift to lower angles with reduced intensity is detected. For concentrations of 3.5 phr and 5 phr, a peak appears with an angle and intensity lower than those of the first peak of Cloisite 20A. For ratios of 8 and 10 phr, two peaks are observed at angles and intensities also lower than the corresponding first and third peaks of Cloisite 20A.

The shift of peaks to smaller angles for concentrations of 5, 8, and 10 phr suggests an expansion of the clay platelets, an increase in interlayer spacing, and penetration of the polymer chains into these layers [16,17]. Therefore, according to X-ray diffraction analysis, Cloisite 20A at concentrations of 1, 2, and 3.5 phr results in mixed structures, with the majority exhibiting exfoliated structures, while concentrations of 5, 8, and 10 phr result in mixed structures where most of the clay is present in an intercalated or conventional composite structure. The addition of Cloisite 30B likely leads to mixed structures across all tested ratios, with the intercalated structure predominating over fully dispersed structures (Figure 1b). In this case, the diffraction peak shows lower intensity as the nanoparticle content decreases and shifts to slightly lower angles (Figure 1b and Table 2).

Nanofil 116 nanoparticles exhibit a peak at a 2θ angle of 7.48° with low intensity. From the X-ray diffraction spectrum (Figure 1c) and Table 2, it is evident that the addition of Nanofil 116 in ratios of 1, 2, 3.5, and 5 phr in polysiloxane shows no peak. This is expected, given the low absorption intensity of Nanofil 116 and its low content in the nanocomposite ratios. Higher ratios of 8 and 10 phr show a peak that shifts slightly to lower angles and decreases in intensity.

The shift of the peaks for the two examined commercial types of montmorillonite (Cloisite 30B and 20A) to lower angles is significantly influenced by the organic modification they undergo through ion exchange, which makes them more organophilic. This modification causes an expansion of the clay platelets in the reinforcing medium, leading to an increase in the gap thickness between the clay layers. This gap expansion facilitates, under appropriate mixing conditions, the penetration of polymer chains into the gaps between the clay platelets and subsequently their dispersion within the polysiloxane matrix. The extent of expansion achieved for nanocomposites with 5, 8, and 10 phr Cloisite 20A/PDMS is greater than that of Cloisite 30B/PDMS nanocomposites, considering that Cloisite 20A is characterized by a larger interlayer spacing. In contrast, the Cloisite 30B/PDMS nanocomposites at these ratios display peaks of lower intensity, a feature associated with the presence of undispersed clay structures. This behavior may be linked to the reduced rate of viscosity increase and the extended vulcanization time observed in these nanocomposites compared to those of Cloisite 20A/PDMS and Nanofil 116/PDMS. Therefore, more time is allowed for the polymer chains to effectively penetrate between the platelets of Cloisite 30B.

### 3.2. Scanning Electron Microscopy (SEM)

**Figure 2:**
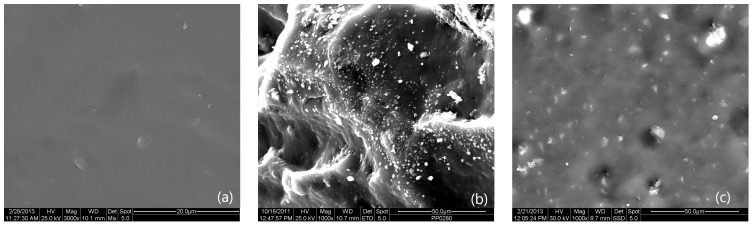
SEM images of: (**a**) PDMS; (**b**) 5 phr Cloisite 20A/PDMS, and (**c**) 5 phr Cloisite 30B/PDMS.

To further study the structure of the polysiloxane nanocomposites, measurements were conducted using scanning electron microscopy (SEM) on sample cross-sections that were gold-coated. Figure 2b,c show the dispersion of the organoclay nanoparticles in PDMS, with agglomerates forming in some areas.

In addition to the processing conditions, the interactions between the clay reinforcement and the PDMS matrix influence the efficiency of dispersion, structure, and relevant properties of the prepared nanocomposites. Cloisite 20A, modified with dimethyl dihydrogenated tallow ammonium chloride, interacts with the hydroxyl-terminated PDMS matrix mainly through van der Waals forces. The higher degree of organic modification of this clay results in increased interlayer spacing between the clay platelets, increasing the available interfacial area for the interaction between the reinforcing medium and elastomeric matrix. Cloisite 30B is modified with methyl, tallow, and bis(2-hydroxyethyl) quaternary ammonium chloride, which introduces hydroxyl groups that can form hydrogen bonds with the hydroxyl groups of the PDMS matrix, promoting interfacial adhesion and intercalation of the PDMS chains into the layered silicate galleries. In addition, the nonpolar backbone of PDMS can interact with the organic modifier of Cloisite 30B through van der Waals forces, contributing to overall compatibility. These interactions promote partial intercalation and exfoliation of the clay structure, improving the dispersion of organoclays in the PDMS matrix. 

### 3.3. Differential Scanning Calorimetry (DSC)

**Figure 3:**
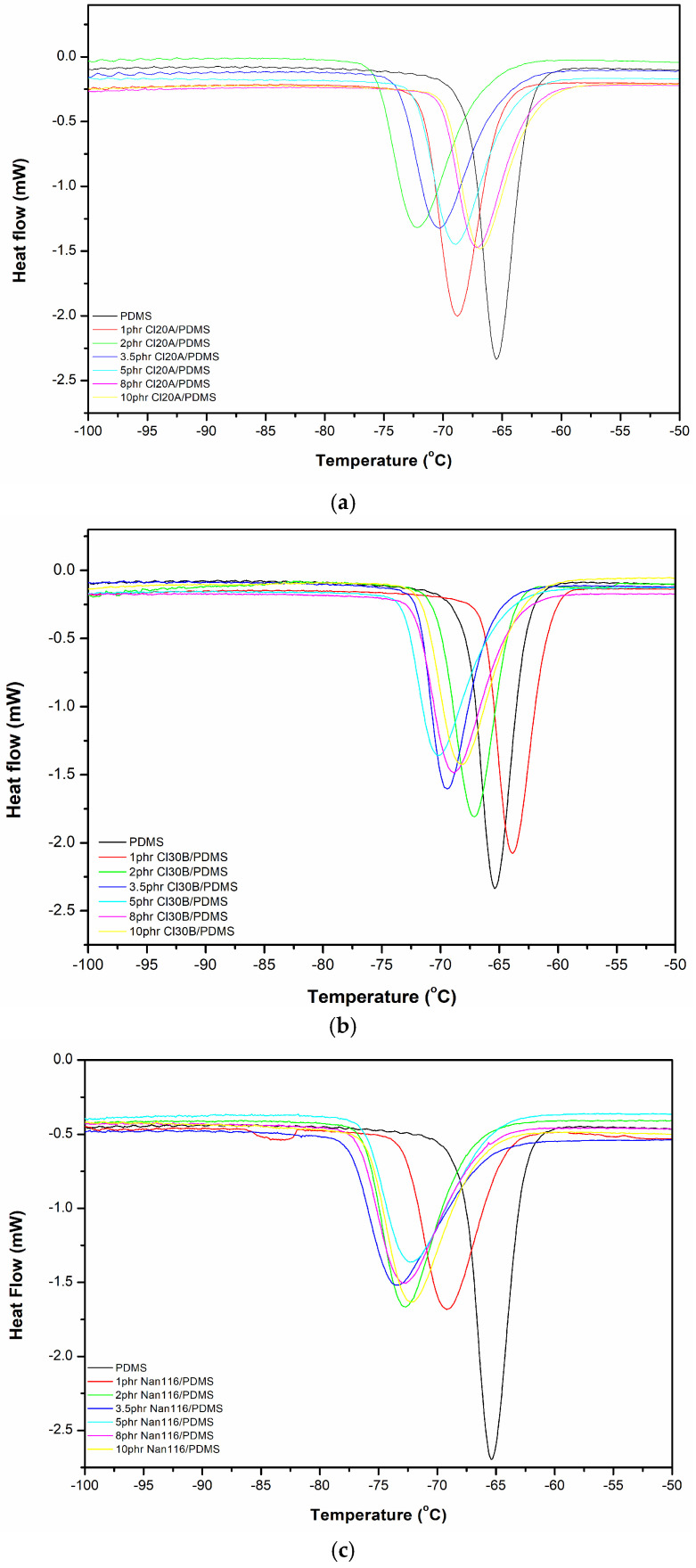
DSC curves of (**a**) Cloisite 20A, (**b**) Cloisite 30B, and (**c**) Nanofil 116/PDMS nanocomposites, during cooling.

From Figure 3, it is evident that the incorporation of both types of organically modified montmorillonite shifts the crystallization peak of unreinforced polysiloxane to the left across all concentrations, resulting in a reduction in the crystallization temperature (*T_c_*) values. The *T_c_* decreases as the amount of Cloisite 20A increases up to 2 phr and that of Cloisite 30B up to 3.5 phr, after which it increases and returns to approximately the levels of the corresponding unreinforced polysiloxane (Table 3). The decrease in *T_c_* in organoclay/PDMS nanocomposites is influenced by the restriction of polymer chain motion due to the presence of dispersed nanoparticles in the elastomeric matrix, which disrupts the crystalline order. Additionally, interactions such as hydrogen bonding and van der Waals forces between the PDMS matrix and organoclays can interfere with the regular packing of polymer chains, resulting in less perfect crystallites and a lower crystallization temperature. 

**Figure 4:**
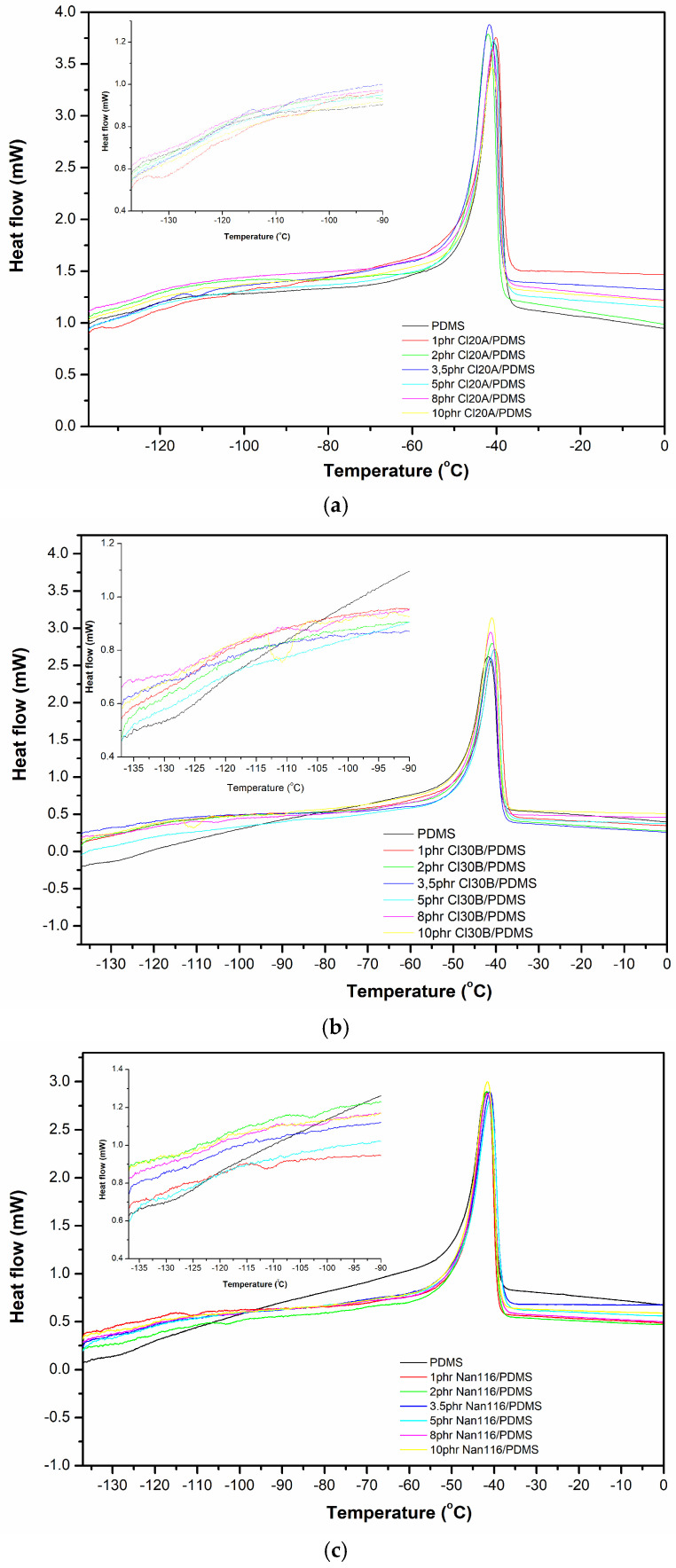
DSC curves of (**a**) Cloisite 20A (**b**), Cloisite 30B, and (**c**) Nanofil 116/PDMS nanocomposites, during heating.

Simultaneously, the crystallization enthalpy (Δ*H_c_*) decreases with an increase in the percentage of both types of modified clay, indicating a reduction in the material’s crystallinity (Table 4). The results in Table 5 show that the glass transition temperature (*T_g_*) of the nanocomposites does not change significantly compared to that of the unreinforced polysiloxane, while a slight decreasing trend was observed in the melting temperature (*T_m_*) with the incorporation of the reinforcing agent (Figure 4). A similar trend is observed for the melting enthalpy (Δ*H_m_*), where a slight decrease is recorded in Table 4.

DSC measurements of polysiloxane nanocomposites with unmodified montmorillonite (Nanofil 116) show that the peak of the “cold” crystallization curve shifts to the left compared to that of unreinforced polysiloxane for all examined ratios (Figure 3c). Meanwhile, the crystallization enthalpy and melting enthalpy remain nearly unchanged across all the ratios of unmodified montmorillonite. The glass transition and melting temperatures of the nanocomposites did not differ from those of the unreinforced polysiloxane. The interfacial interactions between the reinforcement and the polymer matrix affect the mobility of polymer molecules and, consequently, the *T_g_* values of the polymer [18].

A similar effect to that of organoclays was observed for fibers from cotton and polyester textiles when used as reinforcement in PP. The *T_c_* of the PP matrix was significantly higher than that of pure PP due to the nucleating effect of the fibers. Both Δ*H_m_* and Δ*H_c_* notably decreased due to the disruption of the orderly crystalline regions of PP by the fibrous reinforcement [19].

Crystallinity can be calculated from the normalized melting enthalpy using the following equation:

(2) $$X_{c}=\frac{\Delta H_{m}}{\Delta H_{100\%}(1-\varphi )}100\%$$

where *X_c_* is the degree of crystallinity,

Δ*H* is the melting enthalpy of the crystalline regions of the sample

Δ*H*_100%_: the melting enthalpy of a fully crystalline structure (for PDMS, the value from the literature is Δ*H*_100%_ = 61.3 J/g [20]),

*ϕ*: the clay concentration.

The relationship between the melting temperature and crystal thickness is given by the Thompson-Gibbs equation [21]:

(3) $$T_{m}=T_{m}^{0}\left(1-\frac{2\sigma_{e}}{l\Delta H}\right)$$

where $T_{m}^{0}$ is the theoretical crystallization temperature at which the crystal thickness is infinite, $\sigma_{e}$ is the surface energy, *l* is the crystal thickness, and Δ*H* is the melting enthalpy.

From Table 6, it can be observed that the introduction of organically modified montmorillonite reduced the crystallinity percentage in all studied systems, compared to the unreinforced polysiloxane matrix. This behavior is associated with the fact that the dispersed montmorillonite platelets in the polymer matrix act as inhibitors of crystallinity development, restricting the mobility of the elastomer macromolecules. However, the crystal size, as theoretically calculated, remains unaffected. A minor decrease was observed in the case of the unmodified MMT nanocomposites. 

Wang et al. [2] observed that the melting point of silicone nanocomposites increases with the addition of 5 and 10 phr of reinforcing agent but decreases with the addition of 20 phr. This behavior is due to the combination of three factors: (i) The mobility of the polymer chains is reduced due to their incorporation between the layers of the organically modified montmorillonite, leading to an increase in melting temperature. (ii) The dispersion of the reinforcing agent’s layers within the polymer matrix provides crystallization nuclei, which contribute to additional crystallinity. The increased thickness of the crystalline lattice results in an increased melting point. (iii) The melting temperature depends on the lattice density of the silicone/OMMT system. The crosslinking medium can react with both the silicone and the organic modification, leading to incomplete vulcanization, which causes a decrease in *T_m_*. These factors appear to determine the *T_m_* behavior observed in this experimental work.

### 3.4. Thermogravimetric Analysis

**Figure 5:**
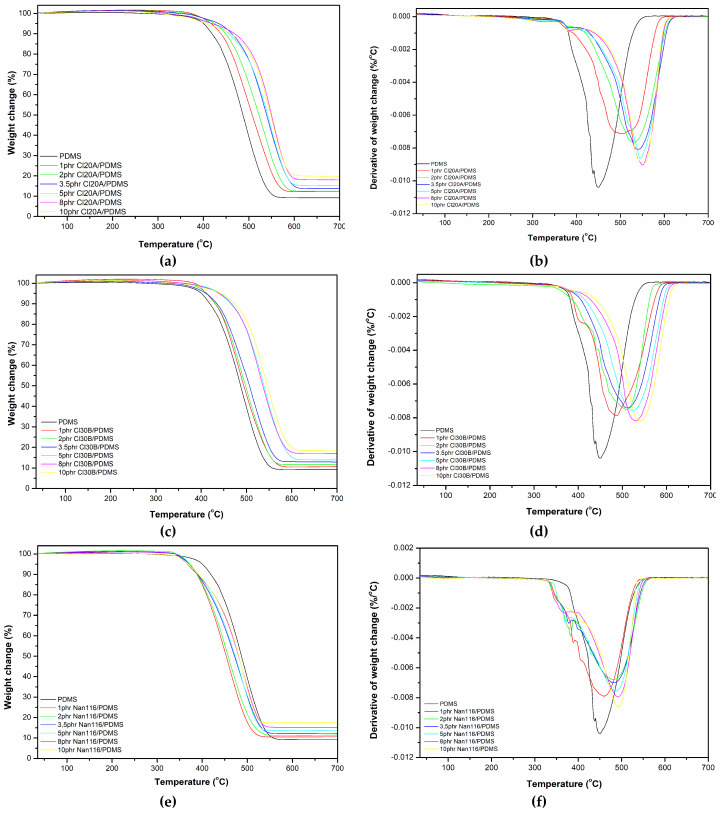
Weight change and derivative of weight change versus temperature of Cloisite 20A (**a**,**b**), Cloisite 30B (**c**,**d**), and Nanofil 116 (**e**,**f**)/PDMS nanocomposites, respectively.

The thermal stabilities of the PDMS and OMMT/PDMS nanocomposites were assessed using TGA. The thermal decomposition of the Cloisite 20A and Cloisite 30B nanocomposites occurs at higher temperatures than that of unreinforced polysiloxane, as shown in Figure 5. 

The TGA spectra results are presented in Table 7 and Table 8, where it is observed that the addition of nanoparticles increases both the onset temperature and the maximum decomposition rate temperature, with further increases noted as the montmorillonite content increases. This enhancement in thermal stability is observed up to a ratio of 8 phr for Cloisite 20A and 8 phr for Cloisite 30B. Subsequently, a downturn in the examined properties is observed, which is associated with the limitation of the effectiveness of the dispersion of the platelets within the elastomer matrix, as determined by the XRD measurements. Finally, an increase in the combustion residue is also observed as the filler content increases (Table 9).

The development of interactions between the dispersed clay platelets and the polysiloxane molecules leads to more stable chemical structures, making their thermal decomposition more difficult. Additionally, within the polymer chains that participate in the intercalated structures of the nanocomposites, protective mechanisms are established that hinder their thermal degradation. The dispersion of exfoliated OMMT platelets hinders both heat transfer and the diffusion of combustion products, potentially leading to the formation of char residues. The formation of a high percentage of fully dispersed nanoclay likely results in a more stable composite structure with higher thermal resistance. The good dispersion of nanoparticles also hinders the introduction of oxygen into the internal layers of the material, which is necessary for thermal decomposition. This fact, combined with protection from heat, results in nanocomposites with more stable thermal decomposition structures.

Table 7 and Table 8 show that the onset decomposition temperature decreases for all examined concentrations of unmodified montmorillonite (Nanofil 116) compared to unreinforced polysiloxane, along with a slight increase in the maximum decomposition rate temperature for all tested ratios. Finally, an increase in the combustion residue is also noted as the filler content increases (Table 9).

There are at least two factors influencing the thermal stability of the composites: (i) the introduction of well-dispersed, organically modified montmorillonite hinders heat transfer, thus improving the thermal stability of the composites. X-ray diffraction analysis indicated that for ratios of 1, 2 and 3.5 phr Cloisite 20A/PDMS, a fully dispersed structure was achieved, as opposed to the intercalated structure observed for Cloisite 30B/PDMS nanocomposites at the same ratios. At higher ratios, Cloisite 30B forms more intercalated structures than Cloisite 20A, although Cloisite 20A may interact more strongly with the silicone matrix. (ii) OMMT contains small molecules that release heat at low temperatures. A high concentration of these molecules reduces the thermal stability of the composites, which explains the decrease in both temperatures at a 10 phr concentration.

Comparing the effects of the two different types of organically modified montmorillonite/polysiloxane composites, it is observed that the incorporation of Cloisite 20A leads to higher values for both examined temperatures compared to the Cloisite 30B nanocomposites. The improvement noted is likely due to the better dispersion of Cloisite 20A platelets compared to those of Cloisite 30B, as evidenced by the X-ray diffraction results. The incorporation of the unmodified reinforcing agent (Nanofil 116) leads to a significant reduction in the onset and maximum decomposition rate temperatures compared to the organically modified clays. In contrast to the good dispersion observed with the organically modified reinforcing agents, the presence of agglomerates in the case of Nanofil 116 results in a degradation of thermal stability. In this case, the MMT nanoparticles act as centers that facilitate the thermal degradation of the polysiloxane.

**Figure 6:**
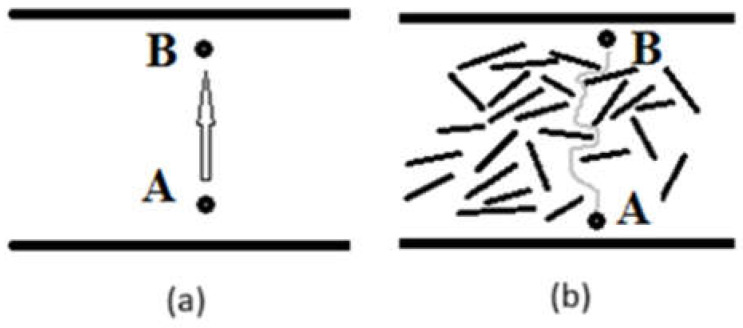
Diffusion models of volatile combustion products: (**a**) unreinforced polysiloxane and (**b**) polysiloxane nanocomposite.

As mentioned, the increase in the onset and maximum decomposition rate temperatures in polysiloxane nanocomposites is also attributed to the difficulty of the volatile combustion products escaping from the composite due to obstruction by the dispersed platelets of the reinforcing agent [3]. Figure 6 illustrates the path taken by the volatile combustion products in unreinforced polysiloxane compared to a nanocomposite of mineral clay. In the unreinforced polysiloxane, the combustion products move directly from point A to point B, whereas the presence of montmorillonite platelets creates a convoluted diffusion pathway that hinders the exit of the products. As a result, the time required for the products to escape in the case of the nanocomposites is longer compared to that of unreinforced polysiloxane.

### 3.5. Kinetic Analysis of Thermal Degradation Through Thermogravimetric Analysis

**Figure 7:**
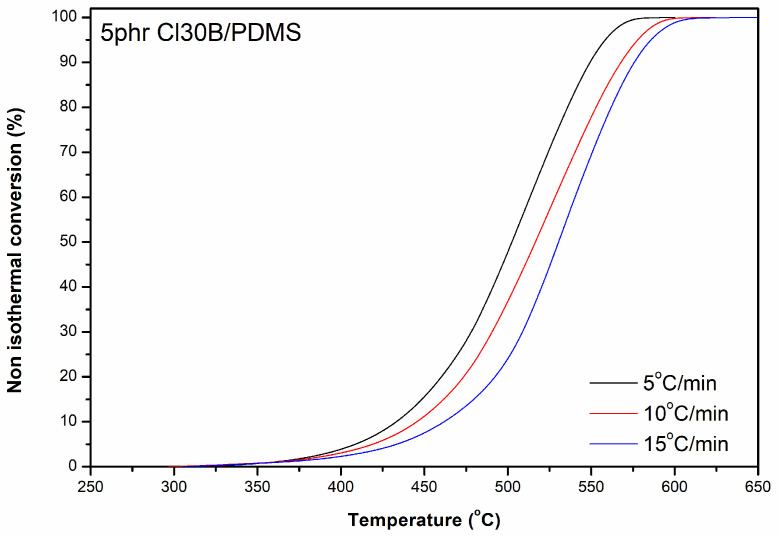
TGA conversion versus temperature curves of 5 phr Cloisite 30B/PDMS nanocomposites, at different heating rates *β*.

The kinetics of thermal degradation can be calculated through dynamic thermogravimetric analysis experiments (Figure 7). In all models of kinetic analysis, the change in conversion with respect to temperature is directly proportional to the change in conversion rate [21]:

(4) $$\frac{da}{dt}=K(T)*f(a)$$

where *f*(a) is the equation that depends on the combustion mechanism. 

*K*(*T*) is given by the Arrhenius equation as follows:

(5) $$K(T)=K_{0}*\exp\left(-\frac{E_{a}}{RT}\right)$$

where *K*_0_ is the pre-exponential factor, 

*E_a_* is the activation energy, and *R* is the universal gas constant. 

In the case of a non-isothermal study and under a constant rate of temperature change, Equation (4) is modified as follows:

(6) $$\frac{da}{dT}=\frac{K_{0}}{\beta }*e^{-\frac{E_{a}}{RT}}f(a)$$

Using the model of the autocatalytic reaction [22], the equation is transformed into

(7) $$\frac{da}{dt}=\left(\frac{1}{\beta }\right)\left(\frac{da}{dT}\right)=\frac{K_{0}}{\beta }*\exp\left(-\frac{E_{a}}{RT}\right)*a^{m}*{\left(1-a\right)}^{n}$$

where *β* = *dT/dt* is the heating rate, and T_max_ is the temperature at which *d*(*d*a/*dt*)/*dt* = 0. 

The activation energy *Ε_α_* can be calculated using the Kissinger equation [23] as follows:

(8) $$E_{\alpha }=-\frac{R\left(d\ln\left(\frac{\beta }{{Τ}_{\max}^{2}}\right)\right)}{\left(d\left(\frac{1}{{Τ}_{\max}}\right)\right)}$$

Alternatively, the activation energy is determined from the slope of the graph of ln(*β*/T_max_^2^) versus 1/T_max_ [24].

**Figure 8:**
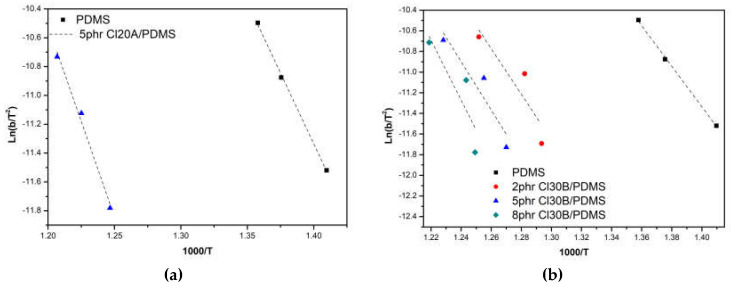
Calculation of activation energy for (**a**) Cloisite 20A and (**b**) Cloisite 30B/PDMS nanocomposites using the Kissinger equation.

To determine the activation energy of combustion through a non-isothermal study, the Kissinger equation was applied. The energy was calculated using the slope of ln(*β*/Τ^2^) − 1000/Τ [24] (Figure 8). The results in Table 10, obtained from the analysis of the straight lines, show that the incorporation of both types of organically modified montmorillonite into the polysiloxane matrix leads to a significant increase in the activation energy for all examined ratios. The most significant increase in energy is observed in the 5 phr Cloisite 20A composites compared to the corresponding Cloisite 30B composites, indicating greater thermal stability provided by the Cloisite 20A nanoparticles. The differentiation observed between the two reinforcing agents is attributed to the better dispersion of Cloisite 20A nanoparticles, as evidenced by the X-ray diffraction results, which provide protective effects and reduce the rate of degradation.

Kim et al. [25] studied the thermal stability of condensation polysiloxane nanocomposites with organically modified montmorillonite using thermogravimetric kinetic analysis of combustion. The study results indicated that the introduction of Cloisite 30B leads to an increase in the activation energy compared to that of the unreinforced elastomer. The activation energy values calculated using the Kissinger equation were very close to those calculated in this study. The improvement in thermal stability was attributed to the terminal hydroxyl groups of Cloisite 30B, which may have participated in the vulcanization reaction, providing greater thermal resistance to the resulting nanocomposite.

According to Camino et al. [26], the activation energy also depends on the heating rate during decomposition. Their results showed that the activation energy values of the studied siloxane range from 54 kJ/mol to 250 kJ/mol, depending on the heating rate. At lower heating rates, the decomposition mechanism can be kinetically controlled, resulting in higher energy values. As the heating rate increases, the focus shifts to the transport of volatile decomposition products from the interior to the exterior of the material.

## 4. Conclusions

Condensation-type polysiloxane composites with montmorillonite of different organic modifications were prepared. To characterize the structure of the montmorillonite nanocomposites, an X-ray diffraction (XRD) study was conducted. The higher degree of organic modification in Cloisite 20A compared to Cloisite 30B results in a larger interlayer spacing between the clay platelets. This facilitates the insertion of elastomer chains between the layers, thereby enabling easy exfoliation and dispersion within the elastomer matrix.

Differential scanning calorimetry (DSC) revealed that the reinforcing agents reduced the cold crystallization temperature of the condensation-type polysiloxane while leaving the glass transition and melting temperatures nearly unaffected. Additionally, the nanocomposites exhibited slightly reduced crystallization and melting enthalpies compared to pure silicone. This behavior occurs because the dispersed montmorillonite platelets inhibit crystallinity development by restricting the mobility of the elastomer macromolecules.

Comparing the effects of different types of montmorillonite (MMT) on the thermal stability of condensation-type polysiloxane during thermogravimetric analysis in an inert environment reveals that Cloisite 20A, compared to Cloisite 30B, provides better thermal stability in all ratios of its nanocomposites with polysiloxane. The unmodified MMT (Nanofil 116) offers improved thermal stability over unreinforced polysiloxane at high reinforcement levels (8 phr); however, in all cases, it lags behind the corresponding nanocomposites with organically modified montmorillonites. The observed effect is likely due to the better dispersion of Cloisite 20A platelets compared to that of Cloisite 30B, as evidenced by the X-ray diffraction results. The development of interfacial interactions between dispersed clay platelets and polysiloxane molecules leads to more stable chemical structures, making thermal decomposition more difficult. In contrast, the presence of agglomerates in the case of unmodified MMT (Nanofil 116) degrades the thermal stability of the PDMS nanocomposites.

## Figures and Tables

**Table 1:** Main characteristics of the organoclays used in this work.

	Cloisite 30B	Cloisite 20A
Organic modifier	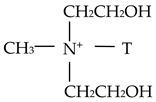 Methyl, tallow, bis-2-hydroxylethyl, quaternary ammonium	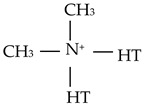 Dimethyl, dihydrogenated tallow, quaternary amonium
Modifier concentration	90 meq/100 g clay	95 meq/100 g clay
Weight loss in ignition	30%	38%

Where: HT is Hydrogenated Tallow (~65% C18, ~30% C16, ~5% C14). T is Tallow (~65% C18, ~30% C16, ~5% C14).

**Table 2:** XRD peak characteristics for the examined types of MMW condensation PDMS nanocomposites.

MMT Content in PDMS (phr)	d (Å)			2θ (^o^)			Intensity		
	Cl20A	Cl30B	Nan116	Cl20A	Cl30B	Nan116	Cl20A	Cl30B	Nan116
1	−	17.60	−	−	5.02	−	−	64.8	−
2	−	18.45	−	−	4.79	−	−	67.6	−
3.5	−	17.60	−	−	5.02	−	−	78.4	−
5	28.46	17.96	−	3.10	4.92	−	183	158	−
8	29.78	17.81	12.06	2.97	4.96	7.33	302	234	62.4
10	30.43	18.23	12.11	2.90	4.84	7.29	432	292	49.3
ΜΜΤ	22.49	17.43	11.82	3.93	5.07	7.48	850	1470	109

**Table 3:** *T_c_* and *T_m_* results for Cloisite 20A, Cloisite 30B, and Nanofil 116/PDMS nanocomposites.

MMT	*T_c_* (°C)			*T_m_* (°C)		
Content	Cl20A	Cl30	Nan116	Cl20A	Cl30	Nan116
0 phr	−65.1 ± 0.9			−46.9 ± 0.3		
1 phr	−64.1 ± 0.6	−60.9 ± 0.2	−65.2 ± 0.3	−47.1 ± 0.0	−45.9 ± 0.6	−47.9 ± 0.3
2 phr	−66.6 ± 0.2	−63.1 ± 1.1	−67.1 ± 0.8	−48.0 ± 0.2	−46.9 ± 0.2	−48.3 ± 0.1
3.5 phr	−65.2 ± 0.1	−65.6 ± 0.1	−66.5 ± 0.5	−47.5 ± 0.3	−47.3 ± 0.3	−48.1 ± 0.3
5 phr	−64.1 ± 0.1	−65.2 ± 0.1	−66.2 ± 0.3	−47.3 ± 0.3	−46.5 ± 0.9	−48.2 ± 0.2
8 phr	−62.8 ± 0.1	−63.6 ± 0.5	−66.5 ± 0.0	−46.9 ± 0.2	−47.0 ± 0.1	−48.2 ± 0.3
10 phr	−62.7 ± 0.4	−63.1 ± 0.3	−66.8 ± 0.3	−46.4 ± 0.9	−46.7 ± 0.3	−48.2 ± 0.1

**Table 4:** Crystallization and melting enthalpy results for Cloisite 20A, Cloisite 30B, and Nanofil 116/PDMS nanocomposites.

MMT	Δ*H_c_* (J/g)			Δ*H_m_* (J/g)		
Content	Cl20A	Cl30	Nan116	Cl20A	Cl30	Nan116
0 phr	25.47 ± 0.64			26.41 ± 0.35		
1 phr	23.72 ± 0.50	24.40 ± 0.71	26.29 ± 1.29	25.22 ± 0.04	25.39 ± 0.07	26.79 ± 0.96
2 phr	23.71 ± 0.30	23.80 ± 0.69	25.43 ± 0.16	26.58 ± 0.27	24.96 ± 0.01	26.67 ± 0.08
3.5 phr	23.74 ± 1.41	23.28 ± 0.32	26.20 ± 2.31	25.76 ± 0.57	24.42 ± 0.10	25.49 ± 0.63
5 phr	23.97 ± 0.51	22.72 ± 0.16	25.19 ± 1.05	27.41 ± 1.56	23.47 ± 0.16	26.67 ± 0.47
8 phr	24.11 ± 0.44	22.72 ± 0.58	25.28 ± 0.45	27.64 ± 0.11	22.96 ± 0.81	26.54 ± 0.16
10 phr	24.11 ± 1.00	21.67 ± 0.79	25.84 ± 1.43	27.21 ± 1.25	23.60 ± 1.35	26.19 ± 1.52

**Table 5:** *T_g_* results for Cloisite 20A, Cloisite 30B, and Nanofil 116/PDMS nanocomposites.

MMT Content	*T_g_* (°C)		
in PDMS	Cl20A	Cl30	Nan116
0 phr	−123.4 ± 0.6		
1 phr	−122.7 ± 1.5	−123.3 ± 1.2	−122.1 ± 0.5
2 phr	−122.2 ± 0.3	−123.5 ± 2.0	−120.9 ± 0.1
3.5 phr	−122.0 ± 1.5	−123.4 ± 0.5	−122.5 ± 0.7
5 phr	−122.1 ± 1.2	−122.3 ± 4.0	−123.6 ± 1.0
8 phr	−122.0 ± 2.2	−121.8 ± 0.8	−121.5 ± 0.5
10 phr	−123.3 ± 2.1	−122.4 ± 0.8	−121.5 ± 1.0

**Table 6:** Crystallinity percentage and crystal thickness results for Cloisite 20A, Cloisite 30B, and Nanofil 116/PDMS nanocomposites.

MMT Content	Cloisite 20A		Cloisite 30B		Nanofil 116	
	Crystallinity (%)	Crystal Thickness (nm)	Crystallinity (%)	Crystal Thickness (nm)	Crystallinity (%)	Crystal Thickness (nm)
0 phr	45.82	465	45.82	465	45.82	465
1 phr	41.14	464	41.97	466	43.70	463
2 phr	43.35	463	41.81	464	43.51	462
3.5 phr	42.03	463	41.75	464	41.58	463
5 phr	44.72	464	40.96	465	43.51	462
8 phr	45.09	464	41.82	464	43.30	462
10 phr	44.38	465	44.28	465	42.73	462

**Table 7:** T_onset_ values of PDMS nanocomposites.

MMT Content	T_onset_ [°C]		
	Cloisite 20A	Cloisite 30B	Nanofil 116
0 phr	420.0 ± 0.3		
1 phr	440.4 ± 2.6	434.7 ± 2.6	391.2 ± 2.1
2 phr	464.6 ± 1.4	436.2 ± 0.4	395.4 ± 2.1
3.5 phr	480.0 ± 1.3	446.7 ± 1.3	405.0 ± 2.4
5 phr	485.1 ± 1.0	459.8 ± 2.1	407.1 ± 1.9
8 phr	498.9 ± 1.1	476.7 ± 0.4	419.1 ± 2.7
10 phr	485.5 ± 1.5	468.5 ± 0.5	423.8 ± 4.5

**Table 8:** T_peak_ values of PDMS nanocomposites.

MMT Content	T_peak_ [°C]		
	Cloisite 20A	Cloisite 30B	Nanofil 116
0 phr	455.4 ± 0.9		
1 phr	522.2 ± 2.1	491.2 ± 1.8	459.5 ± 1.1
2 phr	530.0 ± 3.7	506.9 ± 1.3	465.0 ± 2.8
3.5 phr	543.1 ± 1.1	513.2 ± 2.3	483.1 ± 1.6
5 phr	550.0 ± 4.3	523.7 ± 2.6	488.2 ± 1.9
8 phr	551.9 ± 0.7	531.5 ± 0.7	495.5 ± 0.8
10 phr	536.6 ± 1.6	507.1 ± 1.5	492.6 ± 3.4

**Table 9:** Residue of PDMS nanocomposites.

MMT Content	Residue [%]		
	Cloisite 20A	Cloisite 30B	Nanofil 116
0 phr	9.45 ± 0.33		
1 phr	12.10 ± 3.84	10.39 ± 0.38	10.42 ± 0.25
2 phr	12.42 ± 0.65	12.14 ± 0.51	11.11 ± 0.48
3.5 phr	13.83 ± 0.14	12.92 ± 0.04	12.48 ± 0.34
5 phr	15.03 ± 0.13	13.92 ± 0.27	13.20 ± 0.56
8 phr	18.04 ± 0.06	17.27 ± 1.54	15.02 ± 0.34
10 phr	20.07 ± 0.04	18.53 ± 0.37	16.81 ± 0.98

**Table 10:** Results of kinetic analysis for Cloisite 20A and Cloisite 30B/PDMS nanocomposites.

OMMT Content	E_a_ (kJ/mol)	
in PDMS	Cloisite 30B	Cloisite 20A
0 phr	163.19	
2 phr	183.31	-
5 phr	194.96	220.27
8 phr	240.29	-

## Data Availability

The original contributions presented in this study are included in the article. Further inquiries can be directed to the corresponding author.
